# Editorial: Cellular and molecular mechanisms of neurotropic viral infection

**DOI:** 10.3389/fncel.2024.1365755

**Published:** 2024-01-16

**Authors:** Sara Salinas, Kristen E. Funk

**Affiliations:** ^1^Pathogenesis and Control of Chronic and Emerging Infections, INSERM, University of Montpellier, Etablissement Français du Sang, Montpellier, France; ^2^Department of Biological Sciences, University of North Carolina at Charlotte, Charlotte, NC, United States

**Keywords:** astrocytes, blood-brain barrier, coronavirus, herpes simplex virus, microglia, neurodegenerative diseases, perineural net, Zika virus

Neurotropic viruses describe those that cross the blood-brain barrier (BBB) to infect cells of the central nervous system (CNS). Neurotropic viral infections can lead to acute deleterious effects but also long-lasting psychiatric, neurocognitive, and neurodegenerative symptoms. This Research Topic comprises five publications, each of which discusses a neurotropic virus and mechanistic insight into acute and post-acute neuronal dysfunction.

Zika virus (ZIKV) is a mosquito-borne flavivirus that became of broad interest to the medical community in 2013, when the virus emerged in the Americas causing increased risk of fetal demise and microcephaly in infected pregnant women (Grant et al., 2022). Early gestational age at the time of ZIKV infection correlates with more severe neurodevelopmental effects (Nielsen-Saines et al., 2019). However, the underlying mechanisms that contribute to less severe neurodevelopmental disorders that occur following ZIKV exposure during late pregnancy and early neonatal periods are less well-understood. In this Research Topic, an original study by Engel et al., titled “*Neonatal Zika virus infection causes transient perineuronal net degradation*” describes the effect of ZIKV infection on perineural net (PNN) development using a neonatal model of infection. Engel et al. show that neonatal infection at postnatal day 1 resulted in reduced PNN formation during acute infection that persisted after infection was cleared. With time the impact on PNN morphology resolved, until there was no difference in infected vs. uninfected mice at 1 year post-infection, in agreement with human clinical data, which has shown resolution of neurodevelopmental abnormalities detected in early infancy (Nielsen-Saines et al., 2019).

A defining characteristic of neurotropic infections is the ability of pathogens to cross the BBB to access the CNS; however, while certain viruses are known to broadly disrupt BBB integrity, other viruses are able to enter the brain without compromising the BBB. Original research by Kaur et al. titled “*Zika virus E protein modulates functions of human brain microvascular endothelial cells and astrocytes: implications on blood-brain barrier properties*” used a cell culture model that recapitulates the human BBB to study the effects of ZIKV structural protein E on human brain microvascular endothelial cells (hBMECs) and progenitor-derived human astrocytes. Their results show that ZIKV E protein modulated both cell types, leading to decreased expression of endothelial cell junction proteins in hBMECs, which are critical for BBB integrity. This degraded the BBB integrity, and in concert with the increased expression of proinflammatory chemokines and cytokines from activated astrocytes, may allow the virus to access and infect other CNS cells as well as increase immunopathology due to the resultant neuroinflammation.

Coronaviruses (CoVs) comprise a family of enveloped, single-stranded RNA viruses that can range in severity from the common cold to the global COVID-19 pandemic. SARS-CoV-2 infection manifests primarily as a respiratory infection; however, many patients experience neurologic and neuromuscular complications during acute and post-acute infection phases (Mao et al., 2020). One challenge to studying SARS-CoV-2 *in vivo* is the inability of the virus to infect rodents. SARS-CoV-2 uses the ACE2 receptor to gain entry into host cells, which differs between human and mice such that the murine ACE2 receptor does not efficiently bind SARS-CoV-2 (Hoffmann et al., 2020). Although mouse-adapted strains of virus and transgenic mice expressing human ACE2 have been developed (Qi and Qin, 2022), much has been learned about the antiviral immune response and the impact on neurologic function by studying natural murine CoVs. In this Research Topic, Syage et al. contribute a review article titled, “*Microglia influence immune responses and restrict neurologic disease in response to central nervous system infection by a neurotropic murine coronavirus*,” which discusses the current understanding of molecular and cellular mechanisms by which microglia, the brain-resident immune cells, contribute to host immune defenses to limit neurotropic damage and promote post-infectious recovery in murine CoV models.

In addition to its impact on the CNS, incidence of Guillain-Barré syndrome in COVID-19 patients suggests that SARS-CoV-2 can impair peripheral nervous system function (Caress et al., 2020; Zhao et al., 2020). To better understand the effect of SARS-CoV-2 on motor neurons, Cappalletti et al. developed an *in vitro* model of human motor neurons differentiated from induced pluripotent stem cells (iPSCs), described in this Research Topic. Their article titled, “*Human motor neurons derived from induced pluripotent stem cells are susceptible to SARS-CoV-2 infection*” shows that SARS-CoV-2 can productively infect human iPSC-derived motor neurons. Although iPSC motor neurons did not show cytopathic effects, SARS-CoV-2 altered expression of genes associated with cell survival and metabolism as well as antiviral and inflammatory responses, which may impact cell function through direct and indirect effects.

Among the most ubiquitous viruses to infect humans is herpes simplex virus 1 (HSV-1). The microbial etiology hypothesis of Alzheimer's disease (AD) has gained support in recent years with increasing evidence strengthening the association between many viral infections and AD (Lotz et al., 2021; Levine et al., 2023). In particular, HSV-1 has been a prime suspect as the causal pathogen contributing to the development of AD, even spurring clinical trials utilizing antivirals as a candidate therapy (Eimer et al., 2018; Readhead et al., 2018; Devanand et al., 2020; Hemmingsson et al., 2021). Although there is robust evidence correlating HSV-1 with increased risk of AD, the molecular and cellular mechanisms by which HSV-1 initiates or accelerates AD pathogenesis are less clear. In this Research Topic, Feng et al. review this literature with their contribution titled, “*Mechanistic insights into the role of herpes simplex virus 1 in Alzheimer's disease*.” This review discusses genetic risk factors that are shared between AD and HSV-1 infection, the impact of HSV-1 on pathogenic protein deposition, the double-edged sword of neuroinflammation, and the effect of HSV-1 on host metabolism.

There is robust evidence that viruses negatively impact brain health due to both direct effects of viral infection and indirect effects from the antiviral immune response. The articles published in this Research Topic highlight the continued need for mechanistic insight into the molecular and cellular factors by which viruses cause neural dysfunction and neurodegeneration in order to identify and develop effective interventions for these disorders.

## Author contributions

SS: Writing – review & editing. KF: Conceptualization, Writing – original draft.
